# Physical activity and osteoarthritis: a consensus study to harmonise self-reporting methods of physical activity across international cohorts

**DOI:** 10.1007/s00296-017-3672-y

**Published:** 2017-02-25

**Authors:** L. S. Gates, K. M. Leyland, S. Sheard, K. Jackson, P. Kelly, L. F. Callahan, R. Pate, E. M. Roos, B. Ainsworth, C. Cooper, C. Foster, J. L. Newton, M. E. Batt, N. K. Arden

**Affiliations:** 1 Arthritis Research UK Centre for Sport, Exercise and Osteoarthritis, University of Oxford, Oxford, UK; 2 Faculty of Health Sciences, University of Southampton, Southampton, UK; 3 Physical Activity for Health Research Centre, University of Edinburgh, Edinburgh, UK; 4 Thurston Arthritis Research Centre, University of North Carolina, Chapel Hill, NC USA; 5 Arnold School of Public Health, University of South Carolina, Columbia, SC USA; 6 Department of Sports Science and Clinical Biomechanics, University of Southern Denmark, Odense, Denmark; 7 School of Nutrition and Health Promotion, Arizona State University, Phoenix, AZ USA; 8 MRC Lifecourse Epidemiology Unit, University of Southampton, Southampton, UK; 9 British Heart Foundation Centre on Population Approaches for Non-Communicable Disease Prevention, University of Oxford, Oxford, UK; 10 Centre for Sports Medicine, Nottingham University Hospitals and Arthritis Research UK Centre for Sport, Exercise and Osteoarthritis, Nottingham, UK

**Keywords:** Physical activity, Osteoarthritis, Consensus, Population cohorts, Metabolic equivalent of task

## Abstract

Physical activity (PA) is increasingly recognised as an important factor within studies of osteoarthritis (OA). However, subjective methods used to assess PA are highly variable and have not been developed for use within studies of OA, which creates difficulties when comparing and interpreting PA data in OA research. The aim of this study was, therefore, to gain expert agreement on the appropriate methods to harmonise PA data among existing population cohorts to enable the investigation of the association of PA and OA. The definition of PA in an OA context and methods of harmonization were established via an international expert consensus meeting and modified Delphi exercise using a geographically diverse committee selected on the basis of individual expertise in physical activity, exercise medicine, and OA. Agreement was met for all aims of study: (1) The use of Metabolic Equivalent of Task (MET) minutes per week (MET-min/week) as a method for harmonising PA variables among cohorts; (2) The determination of methods for treating missing components of MET-min/week calculation; a value will be produced from comparable activities within a representative cohort; (3) Exclusion of the domain of occupation from total MET-min/week; (4) The need for a specific measure of joint loading of an activity in addition to intensity and time, in studies of diseases, such as OA. This study has developed a systematic method to classify and harmonise PA in existing OA cohorts. It also provides minimum requirements for future studies intending to include subjective PA measures.

**Electronic supplementary material:**

The online version of this article (doi:10.1007/s00296-017-3672-y) contains supplementary material, which is available to authorized users.

## Introduction

Osteoarthritis (OA) is a chronic condition of the synovial joint, which includes the progressive degeneration of cartilage and the excess growth of bone, often leading to pain and functional impairment [1]. It is one of the leading causes of global disability, with adult prevalence rates reported between 8.5–22% for symptomatic radiographic knee OA [2–4] and 3.4–8.9% for symptomatic radiographic hip OA [3, 5, 6]. To determine risk factors for this disease, it is necessary to analyse previously collected data from longitudinal population cohorts. While there are some well-established risk factors for hip and knee OA, the relationship between physical activity (PA) and OA is inconsistent. This, in part, may be due to the heterogeneous definition of PA used in cohorts and the lack of differentiation between weight-bearing and non-weight-bearing activity.

PA is defined as any bodily movement that results in energy expenditure and is categorised by domains, including occupation, leisure time, daily living, and active travel. Assessment of PA in cohort studies of OA is usually captured by self-report questionnaires, typically including measures of frequency, intensity, duration, and type [7]. Much of the previous research on PA has been completed within the area of cardiovascular disease and obesity and many of the questionnaires and assessments were developed with such health outcomes in mind. Although observational OA-related cohorts have collected information on PA, the parameters measured differ between studies and the number of domains varies, making comparison and interpretation of results difficult. Equally as important is the lack of an available method to assess the degree of joint loading for different physical activities, which is a known risk factor for OA.

A recent systematic review of observational studies, subject to the above limitations, confirmed the association of injury, obesity, and occupational activity with knee and hip OA; however, increased volume and intensity of PA were found to be a risk factor for OA in four studies and protective in another [8]. Due to the heterogeneity in the definition of PA, meta-analysis was not possible for the risk of PA on OA.

To address the difficulties in comparing heterogeneous aggregate data within epidemiological research, an increasingly popular alternative to meta-analysis is individual participant data (IPD) meta-analysis, where the raw individual level data are used for statistical synthesis [9]. This method allows for using the combined power of multiple international cohort studies to address more complex research questions, such as the association between PA and OA [10–18]. A key element of IPD meta-analysis is the harmonization of the variables, which requires a standard measure of both PA and OA from all the included cohorts. Unfortunately, population-based cohort studies have collected PA data using a variety of validated questionnaires and individual questions, which need to be harmonised to be included in any analysis.

To enable this, a number of issues need to be addressed: PA questionnaires and questions vary between cohorts; not all domains are available in all cohorts; some activities are attributes to varying domains (e.g., walking and cycling is included with sport and recreation in a number of cohorts, yet travel in others); duration, intensity, and frequency of PA are not addressed consistently between cohorts; and PA has not previously been assessed by degree of weight-bearing for use in studies of lower limb OA.

A consensus study, including an expert meeting and Delphi approach, was developed to address these issues. The aims of this study were to:

1. Determine the usability of a common metric (Metabolic equivalent of task [MET]) as a key method for harmonising PA assessments/questions between cohorts, and agree upon specific assumptions required to generate METs for each cohort (objectives 1 and 2).
2. Assess the available domains of PA and establish the appropriate assumptions needed to harmonise information between cohorts (objectives 3 and 4).
3. Evaluate the potential to use of a lower limb OA-specific PA measure within the cohorts, taking weight-bearing into consideration (objective 5).
4. Evaluate the use of national PA guidelines to determine the effect of meeting such recommendations on the association with lower limb OA (objective 6).

## Methods

The process consisted of the following steps

### PA expert committee

A multidisciplinary, geographically diverse expert committee was selected on the basis of individual expertise in PA; exercise medicine and OA and each were invited to participate in developing a PA variable for use in normal population-based cohort studies. The expert committee (*n* = 9 of the listed co-authors) met by video conference link in December 2014.

### Expert consensus meeting

The steering group (consisting of authors LG, KL, and NA) conducted a systematic evaluation of international cohorts containing PA and OA data. Four key issues were identified by the steering group whilst establishing the methods to harmonise PA data between cohorts which had not been adequately addressed in the previous literature. Background topic information, the previous research where applicable and specific factors relating to individual cohorts were provided to experts before the meeting to assist informed decision-making. Each issue was presented and facilitated discussion undertaken until agreement was reached.

### Delphi

If a question was raised within the expert meeting and no agreement could be made, due to either a requirement for further investigation or exploration of cohort data, then these items were addressed within a follow-up Delphi technique to obtain consensus. The Delphi technique, which is a structured process of anonymous iteration [19], consisted of an online questionnaire, which was sent to each expert, following the meeting. In accordance with the previous OARSI Delphi exercises to define OA diagnostic criteria [20], inclusion for measures within each round was based on having ≥60% of the votes. Evidenced-based information was provided to inform each item, and where applicable more in depth data from individual cohorts were provided. Unanimous decision-making was made within the first round.

The aims of the consensus study were addressed via six methodological objectives:

1. Determine the suitability of using METs as a key method for harmonising PA exposure variables between cohorts.
2. Determine methods for treating missing components of MET-min/week calculation.
3. Assess the domains of physical activity: how to treat missing domains.
4. Assess the domains of physical activity: the use of occupation as a PA domain in studies with OA as an outcome.
5. Evaluate the use of an OA-specific PA measure taking weight-bearing vs. non-weight-bearing activity into consideration.
6. Establish if thresholds based on national PA guidelines should be used to investigate the association of PA with OA.

## Results

### Determine the suitability of using METs as a key method for harmonising PA exposure variables between cohorts

#### Background information provided to experts

The steering group proposed using the MET to harmonise PA data across population cohorts, based on the results of the literature search and availability of measures within cohorts. PA data can be converted to MET-min/week using the 2011 Compendium of Physical Activities [21] if METs were not already reported. MET is a physiological measure which expresses the intensity and energy expenditure of an activity, and is defined as the ratio of the rate of energy expended during an activity to the rate of energy expended at rest (e.g., 1 MET is the rate of energy expenditure while at rest) [22]. Standard intensity thresholds have been established for MET values of activities with <3 as light, 3–5.9 as moderate, and ≥6 as vigorous [23]. MET-min/week is calculated by multiplying the MET value of an activity (from a standard Compendium of Physical Activities values [21] by the number of minutes per week an activity is done [24]). METs have been used for recommending activity in adults for chronic disease prevention and health promotion [23].

#### Points for expert discussion

Due to the variability between cohorts in the way PA exposure was reported and the wording of PA questions, the benefits, limitations, and feasibility of converting the questions from each cohort into MET-min/week as the standard unit were proposed as the first discussion point to be addressed within the expert consensus meeting.

### Determine methods for treating missing components of MET-min/week calculation

#### Background information provided to experts

To convert PA exposure data to MET-min/week, the duration, frequency, and intensity of an activity are required. In certain cohorts within the current study, some of these parameters were not collected, and a number of assumptions needed to be made. The previous studies have adopted a standard bout time of 30 min when duration was missing from the physical activity question, but the validity of this assumption is unclear [25].

#### Points for expert discussion

Several common PA questionnaires do not include intensity, frequency, or duration of the activity, preventing a direct calculation of MET-min/week. A possible solution proposed by the steering group was to use a standard intensity MET value when calculating MET-min/week if the terminology included “low”, “moderate”, or “high”, and to use a standard of 30 min when duration was missing. These issues raised a requirement for additional investigation via a further Delphi study.

#### Further investigation recommended

For the Delphi exercise, median duration data for individual activities were prepared by the steering group from the cohorts where these data were available (Supplementary Appendix 1). These data were to be presented for agreement within the Delphi to ascertain agreement on the generalisability of the median durations for each activity. Due to some variation between standard activity times between countries, a proposal was made to use the median durations from the Hertfordshire Cohort Study ; a UK study of 3000 men and women born during the period 1931–1939 [15], for other UK cohorts with missing duration data, and similarly to use the durations from Johnston County Osteoarthritis Study: a US community-based, longitudinal study of approximately 3200 rural Caucasian and African American residents aged 45 and older [11], for US cohorts with missing data. The same method was proposed where frequency was identified as missing in one UK cohort (Supplementary Appendix 2).

### Assess the domains of physical activity: how to treat missing domains

#### Background information provided to experts

In previous research, household activities have been included as an essential domain of PA to determine the minimum amount of PA associated with significantly lower risks of all-cause mortality [26, 27]. Household activities have also been specifically included within PA health guidelines [28]. The importance of household activities is also anticipated in determining the association of PA with lower limb OA, particularly due to the known risks of increased cumulative loads seen in such activities and knee OA [29]. Household activities are largely modifiable and are, therefore, an important consideration for any public health message involving physical activity.

#### Points for expert discussion

A significant number of the population-based cohorts did not ask questions regarding household activities and/or gardening, both of which have been identified as important contributors to daily activity loads. Experts were asked to consider the impact of excluding these domains from the primary physical activity variable, and if there were alternative actions to consider.

### Assess the domains of physical activity: the use of occupation as a PA domain in studies with OA as an outcome

#### Background information provided to experts

Occupation, in particular manual labour occupations, is a well-established risk factor for OA [30, 31], and is, therefore, an important consideration in an analysis using OA as the primary outcome. The occupation section within the Compendium of Physical Activities [21] was designed for counting different tasks within one occupation over 1 day and is of limited use for providing an “average” MET value for a specific occupation (i.e., MET values of individual tasks which make up a complete occupation are given and are expected to be done for an entire hour). Occupation is the least modifiable domain of PA and approximately 50% of the consortium cohorts had no or limited occupation data available. It would, therefore, be difficult to calculate a valid exposure in MET-min/week from the available data. The potential for occupation activities to be overestimated is greater than for any other domain, particularly because the occupation MET value within the Compendium of Physical Activities assumes that a specific MET activity is completed for entire hour. To multiply this by hours, worked in 1 week would potentially overestimate weekly METs. The majority of consortium cohorts, which did have occupation data available, did not define tasks within occupation in anything less than 1 day.

#### Points for expert discussion

Experts were asked to discuss the limitations of excluding occupation from the overall physical activity variable specific to studies using osteoarthritis as an outcome. The preferred treatment of an osteoarthritis variable (e.g., MET-min/week and categories) was also explored.

#### Further investigation recommended

A method was required for categorisation into levels, defining occupations into manual and non-manual tasks. Due to the variety of occupation-related tasks available in each cohort, the occupation level reported in the Physical Activity Scale for the Elderly (PASE) questionnaire was used to facilitate the categorisation of occupation-related tasks. The PASE questionnaire is designed to assess the duration, frequency, exertion level, and amount of physical activity undertaken over a 7 day period [32]. The steering group matched the PASE occupation levels to the corresponding four levels of occupation, which were suggested in the expert consensus meeting. The selection of occupation-related tasks with each of the four levels of occupation (sedentary, light, light manual and heavy manual) was prepared to be presented within the follow-up Delphi exercise (Supplementary Appendix 3).

### Evaluate the use of an OA-specific PA measure taking weight-bearing vs. non-weight-bearing activity into consideration

#### Background information provided to experts

OA is at least in part a mechanically driven disease [33]. The association between PA and risk of OA may, therefore, be dependent on joint loading and type of PA [34].

#### Points for expert discussion

There was a need to identify a method that could account for the weight-bearing component of PA. The fifth proposed discussion point for the meeting was, therefore, the potential to use previously established bone and joint loading questionnaires to quantify loading [35, 36].

#### Further investigation recommended

Informed by these decisions, each activity listed within the study cohorts was placed into corresponding loading categories of low, moderate, and high joint loading based on the degree of impact and loading (Supplementary Appendix 4). These were prepared by the steering group for presentation within a follow-up Delphi exercise.

### Establish if thresholds based on national PA guidelines should be used to investigate the association of PA with OA

#### Background information provided to experts

It is important to ensure that a translatable public health message is provided; however, the relationship of meeting or not meeting PA guidelines and the risk of OA are unknown. The use of a threshold based on national guidelines such as those from the American College of Sports Medicine and the American Heart Association [37], the US Physical Activity Guidelines Advisory Committee (US PAGAC) report [23], and the U.K. Department of Health [38] (150 min/week of moderate-equivalent activity) was, therefore, proposed to investigate the effect of meeting current guidelines on the risk of OA.

An additional threshold was suggested to investigate the risk of inactivity on risk of OA; however, there is no global consensus on a defined threshold for inactivity. According to the US PAGAC report [23], <10 min/week of moderate activity is defined as ‘inactive’ and this is in comparison to the UK definition of ‘inactivity’ of <30 min/week moderate activity.

There is growing evidence that increasing steps per day provides health benefits [39–42]. Although originally based on minimal evidence, the 10,000 steps a day guideline now provides a translatable and applicable PA recommendation [43].

#### Points for expert discussion

The final points proposed for discussion within the expert meeting were the use of current national guidelines as a threshold for PA in the investigation of the PA and OA. Also discussed was the use of either a previous arbitrary threshold for inactivity or a new data driven threshold, and the use of steps per day as an equivalent for the translatable outcome instead of 150 min of weekly moderate-equivalent activity.

#### Expert consensus meeting results

Within the expert meeting, each aim was discussed until consensus reached. Where new questions were raised, these were addressed within a follow-up Delphi exercise. There was unanimous agreement for every objective within the consensus meeting and follow-up Delphi. In summary, key agreements were made based upon: the use of MET-min/week as a method for harmonising PA variables between cohorts; defining methods for treating missing components of MET-min/week calculation, in particular the use of a value produced from comparable activities within a representative cohort; the exclusion of the domain of ‘occupation’ from total MET-min/week; and the need for a specific measure of ‘joint loading’ of an activity in studies of bone diseases, such as osteoarthritis.

Details of the decisions made within the consensus meeting and follow-up Delphi are shown in Tables 1 and 2.

**Table 1:** Decisions made within the expert consensus meeting

Objectives	Consensus
1. Determine the suitability of using METs as a key method for harmonising variables between cohorts	To define PA in OA-related cohorts, data will be converted to MET-min/week using the duration, frequency, and type of PA matched to the corresponding MET within the Compendium of Physical Activities [21]. Where METs are already calculated based on Compendium versions prior to 2011, these will be converted to current 2011 Compendium of Physical Activity MET scores
2. Determine methods for treating missing components of MET-min/week calculation	Duration and frequency A standard 30 min assumption for missing duration data was felt to be rather arbitrary, particularly as there are specific activities where the time of the activity will not reflect this duration (e.g., football 90 min, golf 4 h). If a parameter of PA such as duration or frequency is missing, values will be assigned using data derived from nationally representative cohorts. This study has provided average durations (Supplementary Appendix 1) and frequencies (Supplementary Appendix 2) representative of a wide range of activities from both US and UK cohorts to be used when such parameters are missing Intensity Where the parameter of intensity is not measured “moderate” or “general” intensity shall be used for the given activity according to Compendium of Physical Activities. For example: Where intensity of walking is not given, assume standard walking intensity of 3.5 METs Where intensity of bicycling is not given, assume bicycling intensity of 7.5 METs Where there is no differentiation between walking and cycling (how long you ‘walk or cycle’), assume a MET value of 5.5 Type Where a list of examples is given and one single type of activity cannot be chosen, assume MET based on the average of all of the examples, e.g., light sport (such as bowling, golf with a cart, shuffleboard, and fishing). Average of these type examples = 3.3 METs according to the Compendium of Physical Activities 2011 [21] Time period When a cohort questionnaire asked about ‘last week’, assume that this is a typical week. When asked about months per year and times per month, calculate an average week over entire year. When asked about times per month, divide by four for times per week Walking and cycling Where walking or cycling has a separate domain, disregard any walking or cycling equivalent variable that is also noted in sport and recreation domain
3. Assess the domains of physical activity: how to treat missing domains	Without these domains, it was believed there may be an underestimation in PA within these cohorts. Available household data are good quality; therefore, it was decided to impute missing data when statistically possible
4. Assess the domains of physical activity: the use of occupation as a PA domain in studies with OA as an outcome	It was agreed that occupation will not be included as a domain when calculating weekly METs. Instead results will be stratified by levels of occupational activity Findings from the consensus meeting revealed a similar international technical consensus meeting and PA initiative recently began refining working categories into the following levels: heavy manual, light manual, light, and sedentary (Kelly P: Personal communication). Occupations and occupation-related tasks, which are commonly used within population cohort studies, have been categorised into these four levels of occupation, according to the PASE questionnaire, which can be used to stratify results (Supplementary Appendix 3)
5. Evaluate the use of an OA-specific PA measure taking weight-bearing vs. non-weight-bearing activity into consideration	Agreement was made that the degree of weight-bearing or joint loading should be considered on a scale. A decision was made within the expert meeting for the working group to further assess the use of a joint loading type questionnaires and use findings to inform further expert decision-making within the subsequent Delphi exercise It was proposed to use the joint impact and torsional load categorization, developed by Buckwalter and Lane [35] as an example to classify activities into low, moderate, or high levels of impact/torsional load. This was chosen particularly for its relevance to OA, having been used by other researchers looking at the relationship between joint loading activity and OA Activities within the study cohorts were then categorised according to these classifications. Any activities that were not described by Buckwalter and Lane [35] were evaluated by the experts and placed in comparable categories. Agreement was reached within the Delphi exercise for the joint loading categories of low, moderate, and high and the activities placed within each category (see Supplementary Appendix 4 for table provided to experts)
6. Establish if thresholds based on national PA guidelines should be used to investigate the association of PA with OA	Agreement was made to evaluate the dose–response of METs against risk of OA to provide data driven thresholds to define ‘inactivity’ or ‘insufficient activity’, rather than use an arbitrary threshold. The primary analysis will exclude occupational METs and a secondary analysis will use overall METs from all domains if possible Agreement that a guideline based on the number of steps per day needed to reduce the risk of OA would be a valuable metric for some people to measure their PA levels, although this would require further assumptions to be made in the process of converting MET values to steps. An attempt will be made to complete the conversion based on results, providing that there is an inflection point for the reduced risk of OA

**Table 2:** Modified Delphi results (questions were based on evidence and supplementary information provided in Appendices 1–4)

Questions	Agreement (%) (*n* = 9)
1. Do you agree with the use of the levels of occupational activities, suggested by a previous consensus group, within the current study? (sedentary, light, light manual, and heavy manual)	100
2. Do you agree with the selected occupation-related tasks within each of the levels of occupation? (see Appendix 3 for list of occupation-related tasks)	
Sedentary	100
Light	67
Light manual	78
Heavy manual	67
3. Do you agree with the activity joint loading categories and definitions? (see Appendix 4 for list of activities)	
Low	78
Moderate	78
High	78
4. Do you agree with the average durations that have been assigned to the listed activities? (see Appendix 1 for median durations of activities)	89
5. For these duration values, should we use the absolute median number or round it up to the nearest 15 minutes?	67
6. When frequency is missing an assumption will made based on individual activity data from a matched cohort. Do you agree with this assumption? (see Appendix 2 for average frequencies of activities)	67

## Discussion

Our research describes a method to classify and harmonise PA data for epidemiology research in population-based OA-related cohorts, based on international consensus. The recommendations will allow the IPD meta-analysis of PA and incident lower limb OA to be undertaken using the most comprehensive PA data possible. In addition, it will be useful not only for future epidemiology research into OA that uses physical activity and IPD studies, but also to guide researchers planning to collect PA data for current or future epidemiological research.

Recommendations for combining data between cohorts, based on expert opinion in this study, are to use MET-min/week as a standardised measure of PA. In the instance of a missing parameter, such as duration, frequency, or intensity, the methods of assigning values using data derived from representative cohorts or the Compendium of Physical Activities have been agreed. The likely effects of occupation should be accounted for by categorisation of occupation type and stratification. The weight-bearing aspect within PA should be taken into consideration when using OA as an outcome.

Although national PA guidelines are an essential and translatable source of health prescription, they were not designed with OA in mind. Guidelines are required to address health and, therefore, recommendations relative to OA, an increasing global burden, are required to add to other disease areas. There are a number of domains of PA to consider when assessing the target for meeting national recommendations; these include leisure time, household/gardening, active travel, and occupational activities. These domains are particularly pertinent when considering the effect of PA on lower limb OA due to the weight-bearing nature of many activities within each and the difficulty is that there is currently no index to combine the physiological measure of METs and joint load.

An increasingly popular alternative to meeting 150 min/week of moderate-equivalent activity is the daily accumulation of 10,000 steps. Experts agreed that this could be a useful method of assessing PA against OA in the future. Recommendations from the expert consensus study suggest that a guideline based on the personalised optimum number of steps per day to reduce the risk of OA would be a valuable public health message.

A limitation of this study was that decisions had to be based on already data that were already available, because due to the requirement to use existing population-based cohorts to investigate the association between PA and OA. There are also known limitations for using METs as a measure of PA, particularly when making comparisons among a number of studies or populations. Studies have previously measured METs based on a varying numbers of activities from the total available from the domains of PA [44]. As a MET is the total volume of a given activity, which combines frequency, intensity, and duration, the effect of duration or intensity alone cannot be deciphered. IPD meta-analysis allows for the use of original raw data, so that all aspects of PA can be included and where no available in certain circumstances imputation methods can be applied. Likewise, the access to original data allows for data driven thresholds to derived and provides potential for observing individual parameters of PA, be it intensity or duration.

A further limitation to this study was the use of PA data from more than one decade ago to calculate median duration for those cohorts missing this data. Although PA levels are likely to have changed, since these data were collected the committee felt, this was still a more appropriate representation of duration than using a standard 30 min, which is likely to over or underestimate activity levels.

Our study provides the first expert consensus on the limitations of and the methods for harmonising PA data in population-based OA cohort studies. The application of these recommendations in future individual patient meta-analysis on PA and OA will provide a homogeneous way to assess PA in cohorts from around the world. It will also allow for quantifying the volume of PA and examine the shape of the dose–response curve for PA and OA as well as the ability to apply new thresholds for future national PA guidelines. These findings will also be useful for any study investigating PA and other long-term health outcomes in existing cohort data. The recommendations arising from this consensus study for the collection of PA data in normal population-based cohort studies are: the need for all parameters of a given activity (duration, frequency, and type/intensity) within a specified timeframe; PA measured in all domains of daily life (sport/leisure, household/gardening, active travel, and occupation); an occupation measure which can be used to calculate accurate MET-min/week value in addition to a manual labour (occupational tasks) in terms of OA risk; and a measure of joint loading for each reported activity.

## Electronic supplementary material

Below is the link to the electronic supplementary material.

Supplementary material 1 (DOCX 18 KB)

Supplementary material 2 (DOCX 15 KB)

Supplementary material 3 (DOCX 13 KB)

Supplementary material 4 (DOCX 15 KB)
