# Supplementary material for: Physical activity and osteoarthritis: a consensus study to harmonise self-reporting methods of physical activity across international cohorts
Source: Rheumatol Int. 2017 Feb 25;37(4):469–78. doi: 10.1007/s00296-017-3672-y (PMC5357277; doi:10.1007/s00296-017-3672-y)
Supplement: Supplementary file 1 — Supplementary material 1 (DOCX 18 KB) [file 296_2017_3672_MOESM1_ESM.docx]

Appendix 1. PA median duration in minutes for a UK and US cohort

| **From US data set** | | **From UK data set** | |
| --- | --- | --- | --- |
| **Activity name** | **Median minutes** | **Activity name** | **Median minutes** |
| **LAWN AND GARDEN ACTIVITIES** | | | |
| Mowing lawn with riding mower | 90 | Mowing lawn riding mower | 80 |
| Mowing walking behind power mower | 60 | Mowing lawn with hand or power mower | 60 |
| Mowing lawn with push mower | 60 | Planting seeds, shrubs, flowers | 60 |
| Weeding and cultivating garden | 60 | Weeding, cultivating garden, trimming shrubs or trees | 60 |
| Spading, digging, filling in garden | 60 | Digging, spading, filling garden, composting | 60 |
| Raking lawn | 60 | Raking lawn | 37.5 |
| Snow shoveling by hand | 45 | Sacking grass, leaves | 30 |
| **HOME REPAIR ACTIVITIES** | | | |
| Carpentry in workshop | 120 | Carpentry inside | 90 |
| Painting inside or hanging wallpaper | 210 | Painting indoors, wallpapering, plastering, scraping, waxing floors | 210 |
| Carpentry outside | 180 | Carpentry, outside house -erecting shed, fences, laying patios etc. | 121 |
| Painting outside of house | 180 | Painting outdoors, guttering, fascias, windows etc. | 180 |
|  |  | Plumbing and/or wiring | 120 |
| **HOUSEHOLD ACTIVITIES** | | | |
|  |  | Shopping | 90 |
| Heavy housework | 60 | General household cleaning | 60 |
| Light housework | 60 | Vacuuming and mopping | 30 |
|  |  | Cooking or food preparation, putting away groceries | 60 |
|  |  | Scrubbing floors on hands & knees, scrubbing bathroom | 20 |
|  |  | Ironing | 60 |
| **MISCELLANEOUS** | | | |
|  |  | Playing a musical instrument | 30 |
|  |  | Child care - dressing, bathing, grooming, feeding, occasional lifting | 90 |
|  |  | Sitting playing with child - light effort | 60 |
|  |  | Running/walking to play with child - moderate effort | 60 |
|  |  | Elderly or disabled adult care (lifting, dressing, bathing, grooming etc.) | 105 |
| **WALKING** | | | |
| Walking for pleasure | 30 | Walking for pleasure outdoors | 60 |
| Walking to work | 22.5 | Walking to and/or from work | 30 |
| Using stairs when elevator is available | 5 | Walking upstairs, or climbing a ladder | 1 |
|  |  | Walking indoors | 70 |
| Cross country hiking | 180 | Cross-country hiking | 120 |
| Back packing | 165 | Backpacking | 240 |
| Mountain climbing | 180 | Mountain or rock climbing | 210 |
| **CYCLING** | | | |
| Bicycling to work and/or for pleasure | 30 | Bicycling outdoors | 60 |
| **DANCING** | | | |
| Dancing-ballroom, square and/or disco | 105 | Slow dancing- slow ballroom, waltz, foxtrot | 60 |
| Dancing, aerobic, ballet | 45 | Fast dancing - fast ballroom, disco, folk, line, square, country | 60 |
| Horseback riding | 90 | Horse riding | 75 |
| **FISHING AND HUNTING** | | | |
| Fishing from river bank | 240 | Fishing from the river bank | 300 |
| Fishing in stream with wading boots | 360 | Fishing in a stream, in waders | 480 |
| Hunting pheasants or grouse | 240 | Hunting general (including large or small game) | 360 |
| Hunting rabbits/chickens/squirrel/raccoon | 240 | Shooting game or trap shooting -duck grouse etc. | 450 |
| Hunting large game: deer, elk, bear | 240 |  |  |
| **GOLF** | | | |
| Riding a power cart | 240 | Golf riding a power cart | 240 |
| Walking, pulling clubs on cart | 225 |  |  |
| Walking and carrying clubs | 240 | Golf carrying own clubs | 240 |
| **SPORTS** | | | |
| Bowling | 120 | Bowling | 120 |
| Volleyball | 90 |  |  |
| Table tennis | 60 | Table tennis, ping pong | 30 |
| Tennis, singles | 120 | Tennis | 60 |
| Tennis, doubles | 120 |  |  |
| Softball | 60 |  |  |
| Badminton | 60 | Badminton | 90 |
| Paddle ball | 45 |  |  |
| Racket ball | 105 |  |  |
| Basketball: non-game | 60 |  |  |
| Basketball: officiating | 120 |  |  |
| Touch football | 120 |  |  |
| Handball |  |  |  |
| Squash |  | Squash | 37.5 |
| Soccer | 105 | Football | 75 |
|  |  | Cricket | 160 |
|  |  | Darts | 60 |
| **CONDITIONING EXERCISES** | | | |
|  |  | Light/moderate effort exercises-light aerobics, home exercises | 15 |
|  |  | Vigorous effort exercises, push-ups, sit-ups, pull-ups | 15 |
| Home exercise | 20 | Yoga, stretching exercises, Pilates | 60 |
| Health club exercise | 60 | Gym club workout - moderate effort, general gym workout | 60 |
| Jog/walk combination | 30 | Jogging | 15 |
| Running | 30 | Running light effort outdoors &/or treadmill | 15 |
|  |  | Running vigorous effort more than 7mph outdoors &/or treadmill | 15 |
| Weight lifting | 20 | Lifting weights/loads-any loads, light/moderate effort | 15 |
| **WATER ACTIVITIES** | | | |
| Water skiing | 30 | Water-skiing |  |
| Sailing in competition | 180 | Sailing for pleasure - boat & board sailing, windsurfing, ice sailing | 240 |
| Canoeing or rowing for pleasure | 150 | Rowing or canoeing for pleasure (not including rowing at the gym) | 30 |
| Canoeing or rowing for competition |  |  |  |
| Canoeing on a camping trip | 30 |  |  |
| Swimming (at least 50 ft.) at a pool | 60 | Swimming-laps, lane, freestyle, slow, moderate or light effort | 30 |
| Swimming at the beach | 60 | Swimming-leisurely, inc. seaside swimming but not lap swimming | 30 |
|  |  | Swimming-laps, lane, freestyle, fast, vigorous effort | 20 |
| Scuba diving | 90 | Scuba diving | 30 |
| Snorkeling | 90 | Snorkeling | 30 |
| **WINTER ACTIVITIES** | | | |
| Snow skiing, downhill | 300 | Skiing | 180 |
| Snow skiing, cross country |  |  |  |
| Ice (or roller) skating | 120 | Ice or roller skating | 150 |
| Sledding or tobogganing | 120 |  |  |
| **OTHER ACTIVITIES** | | | |
| Exercise in bed | 10 |  |  |
