# Supplementary material for: Physical activity and osteoarthritis: a consensus study to harmonise self-reporting methods of physical activity across international cohorts
Source: Rheumatol Int. 2017 Feb 25;37(4):469–78. doi: 10.1007/s00296-017-3672-y (PMC5357277; doi:10.1007/s00296-017-3672-y)
Supplement: Supplementary file 2 — Supplementary material 2 (DOCX 15 KB) [file 296_2017_3672_MOESM2_ESM.docx]

Appendix 2. PA frequencies (number of times per month) from a US nationally matched cohort

| **WALKING AND MISCELLANEOUS** | |
| --- | --- |
| Walking for pleasure outdoors | 10 |
| Walking to and/or from work | 16 |
| Walking indoors | 30 |
| Walking upstairs, or climbing a ladder | 120 |
| Cross-country hiking | 2 |
| Backpacking | 5 |
| Mountain or rock climbing | 2 |
| Bicycling outdoors | 4 |
| **CONDITIONING EXERCISE** | |
| Light/moderate effort exercises-light aerobics, home exercises | 15 |
| Vigorous effort exercises, push-ups, sit-ups, pull-ups | 8 |
| Yoga, stretching exercises, Pilates | 4 |
| Gym club workout - moderate effort, general gym workout | 5 |
| Jogging | 1 |
| Running light effort outdoors &/or treadmill | 8 |
| Running vigorous effort more than 7mph outdoors &/or treadmill | 8 |
| Lifting weights/loads-any loads, light/moderate effort | 10 |
| **WATER ACTIVITIES** | |
| Water-skiing |  |
| Sailing for pleasure - boat & board sailing, windsurfing, ice sailing | 2.5 |
| Rowing or canoeing for pleasure (not including rowing at the gym) | 3 |
| Swimming-leisurely, inc. seaside swimming but not lap swimming | 4 |
| Swimming-laps, lane, freestyle, slow, moderate or light effort | 4 |
| Swimming-laps, lane, freestyle, fast, vigorous effort | 4 |
| Scuba diving | 2 |
| Snorkeling | 1 |
| **WINTER ACTIVITIES** | |
| Skiing | 5 |
| Ice or roller skating | 4 |
| **SPORTS** | |
| Bowling | 4 |
| Table tennis, ping pong | 2 |
| Tennis | 4 |
| Horse riding | 1 |
| Slow dancing- slow ballroom, waltz, foxtrot | 1 |
| Fast dancing - fast ballroom, disco, folk, line, square, country | 2 |
| Football | 26 |
| Badminton | 4 |
| Cricket | 4 |
| Darts | 4 |
| Squash | 4.5 |
| **GOLF** | |
| Golf carrying own clubs | 4 |
| Golf riding a power cart | 4.5 |
| **LAWN AND GARDEN ACTIVITIES** | |
| Mowing lawn with hand or power mower | 4 |
| Mowing lawn riding mower | 4 |
| Raking lawn | 2 |
| Weeding, cultivating garden, trimming shrubs or trees | 4 |
| Digging, spading, filling garden, composting | 4 |
| Sacking grass, leaves | 4 |
| Planting seeds, shrubs, flowers | 4 |
| **HOME ACTIVITIES** | |
| Shopping | 8 |
| General household cleaning | 15.5 |
| Vacuuming and mopping | 8 |
| Cooking or food preparation, putting away groceries | 30 |
| Scrubbing floors on hands & knees, scrubbing bathroom | 4 |
| Ironing | 8 |
| Playing a musical instrument | 10 |
| Child care - dressing, bathing, grooming, feeding, occasional lifting | 4 |
| Sitting playing with child - light effort | 4 |
| Running/walking to play with child - moderate effort | 4 |
| Elderly or disabled adult care (lifting, dressing, bathing, etc.) | 30 |
| Carpentry, outside house -erecting shed, fences, laying patios etc. | 3 |
| Carpentry inside | 4 |
| Plumbing and/or wiring | 2 |
| Painting indoors, wallpapering, plastering, scraping, waxing floors | 3.5 |
| Painting outdoors, guttering, fascias, windows etc. | 2 |
| **FISHING AND HUNTING** | |
| Fishing from the river bank | 4 |
| Fishing in a stream, in waders | 2 |
| Hunting general (including large or small game) | 4 |
| Shooting game or trap shooting -duck grouse etc. | 2.5 |
