# Supplementary material for: Physical activity and osteoarthritis: a consensus study to harmonise self-reporting methods of physical activity across international cohorts
Source: Rheumatol Int. 2017 Feb 25;37(4):469–78. doi: 10.1007/s00296-017-3672-y (PMC5357277; doi:10.1007/s00296-017-3672-y)
Supplement: Supplementary file 3 — Supplementary material 3 (DOCX 13 KB) [file 296_2017_3672_MOESM3_ESM.docx]

Appendix 3. Levels of occupation defined within previous consensus study, attributed to occupation orientated tasks

| **Levels of occupation** | **PASE occupation orientated tasks (Washburn et al., 1993)** | **cohort occupation related task questions attributable to PASE** |
| --- | --- | --- |
| Sedentary | Mainly sitting with slight arm movements | Sitting |
|  |  | Mainly sitting with slight arm movements |
|  |  | Driving |
| Light | Sitting or standing with some walking | Sitting or standing with some walking |
|  |  | Mostly standing and or walking |
|  |  | Standing |
|  |  | Walking |
|  |  | Handling peddles with feet |
|  |  | Walking on irregular surfaces |
|  |  | Sitting/moving on knees |
|  |  | Walk 2 miles per day |
| Light Manual | Walking, with some handling of materials generally weighing less than 50 pounds | Homemaker |
|  |  | Climbing stairs |
|  |  | Walking, some handling materials less than 50lbs |
|  |  | Moving loads <5kg |
|  |  | Moving loads 10kg+ |
|  |  | Climb 30+ flights of stairs in working day |
|  |  | Kneeling/squatting |
|  |  | Hands below knees |
|  |  | Often squatting |
|  |  | Bending repeatedly |
|  |  | Work at speed determined by a machine |
|  |  | Crawling |
| Heavy Manual | Walking and heavy manual work often requiring handling of materials weighing over 50 pounds | Walking, heavy manual work often handling material > 50lbs |
|  |  | Heavy work standing in one place (e.g. lifting boxes) |
|  |  | Moving loads >25kg |
|  |  | Moving loads >50kg |
|  |  | Moving loads >100kg |
|  |  | Maximal force exertions |
|  |  | Physical hard work |
|  |  | Work with vibrating tools |
|  |  | Farmer |
