# Supplementary material for: Physical activity and osteoarthritis: a consensus study to harmonise self-reporting methods of physical activity across international cohorts
Source: Rheumatol Int. 2017 Feb 25;37(4):469–78. doi: 10.1007/s00296-017-3672-y (PMC5357277; doi:10.1007/s00296-017-3672-y)
Supplement: Supplementary file 4 — Supplementary material 4 (DOCX 15 KB) [file 296_2017_3672_MOESM4_ESM.docx]

Appendix 4. Activity joint loading and impact categories, defined based upon the joint impact and torsional load categorization (Buckwalter & Lane, 1997)

| **Category** | **Definition** | **Example Activities** | |
| --- | --- | --- | --- |
| Low | Low impact + low torsion | Aerobic swimming | Press ups |
|  |  | Archery | Rambling |
|  |  | Ball room dancing | Rowing machine |
|  |  | Basketball shooting Baskets | Sequence dancing |
|  |  | Bell ringing | Shuffle board |
|  |  | Brisk walking | Square dancing |
|  |  | Callanetics (mod) | Swimming (moderate) |
|  |  | Exercise bike | Swimming (high intensity) |
|  |  | Fishing (general) | Swimming (20m/month) |
|  |  | Floor exercises | Swimming laps (hard effort) |
|  |  | Frisbee throwing | Swimming laps (moderate effort) |
|  |  | Gardening | Swimming laps (easy effort) |
|  |  | Gentle exercise | Walking |
|  |  | Golf (with cart) | Walking dog |
|  |  | Golf (no cart) | Water aerobics |
|  |  | Golf (general) | xc ski machine |
|  |  | Housework | Yoga |
|  |  | Hunting (general) |  |
| Moderate | Moderate impact + low torsion  OR  Low impact + high torsion | Keep fit | Jazz dancing |
|  |  | Aerobics | Racquet sports |
|  |  | Backpacking uphill | Riding |
|  |  | Backpacking on level ground | Rowing (general) |
|  |  | Badminton | Sailing |
|  |  | Badminton doubles | Short tennis |
|  |  | Bowling | Skating (roller) |
|  |  | Bowls | Skiing |
|  |  | Cycling (general) | Skiing (downhill) |
|  |  | Cycling (high intensity) | Skiing (cross country) |
|  |  | Dancing | Skiing downhill |
|  |  | Disco dancing | Slimnastics |
|  |  | Exercise/s | Table tennis |
|  |  | Folk dancing | Tennis (doubles) |
|  |  | Gym | Walking/jogging |
|  |  | Gym exercise | Walking (power) |
|  |  | Home exercises | Weight training |
|  |  | Ice skating | Weight lifting |
|  |  | Indoor bowls | Working out |
| High | High impact + low or high torsion | Badminton singles | Jogging |
|  |  | Basketball (in game) | Keep fit exercise |
|  |  | Basket ball | Netball |
|  |  | Circuit training (moderate) | Running |
|  |  | Circuit training (vigorous) | Tennis (singles) |
|  |  | Football (soccer) | Vigorous exercise |
|  |  | Football (in game) | Water skiing |
|  |  | Ultimate Frisbee |  |
